# Perfusion Angiography in Acute Ischemic Stroke

**DOI:** 10.1155/2016/2478324

**Published:** 2016-07-03

**Authors:** Fabien Scalzo, David S. Liebeskind

**Affiliations:** Department of Neurology, University of California, Los Angeles, Los Angeles, CA 90024, USA

## Abstract

Visualization and quantification of blood flow are essential for the diagnosis and treatment evaluation of cerebrovascular diseases. For rapid imaging of the cerebrovasculature, digital subtraction angiography (DSA) remains the gold standard as it offers high spatial resolution. This paper lays out a methodological framework, named perfusion angiography, for the quantitative analysis and visualization of blood flow parameters from DSA images. The parameters, including cerebral blood flow (CBF) and cerebral blood volume (CBV), mean transit time (MTT), time-to-peak (TTP), and *T*_max_, are computed using a bolus tracking method based on the deconvolution of the time-density curve on a pixel-by-pixel basis. The method is tested on 66 acute ischemic stroke patients treated with thrombectomy and/or tissue plasminogen activator (tPA) and also evaluated on an estimation task with known ground truth. This novel imaging tool provides unique insights into flow mechanisms that cannot be observed directly in DSA sequences and might be used to evaluate the impact of endovascular interventions more precisely.

## 1. Introduction

Visualization of blood flow inside brain vessels is essential for the diagnosis and treatment evaluation of cerebrovascular disorders. First attempts date back to the early years of angiography [1], a technique that relies on X-ray imaging of iodinated radioopaque contrast agent previously injected into the blood stream. Over the years, the technique has improved and benefited from the appearance of digital cameras, leading to digital subtraction angiography (DSA) [2–4] which allows for the unwanted elements (e.g., skull) to be removed by image subtraction. Today, DSA remains a central and widely used imaging technique to assess blood flow during neurovascular interventions of stroke, for instance. In practice, several limitations hinder the use of DSA; images are qualitative; they are displayed in grayscale and need to be browsed frame by frame to observe temporal differences. This paper addresses these limitations by presenting a framework, perfusion angiography, for the quantitative analysis and visualization of perfusion and delay parameters from DSA.

The popularity of DSA can be attributed to its good spatiotemporal resolution which is not easily matched by other acquisition techniques such as magnetic resonance imaging (MRI) and computed tomography (CT). Vascular abnormalities such as narrowing, blockage, or malformations can be visualized precisely in DSA. In addition, DSA is minimally invasive and is readily available in interventional suites of modern intensive care units (ICUs). Minimal cost, low risks, and rapid acquisition time are other features in favor of DSA. Although it may be argued that DSA will gradually be replaced in the future by CT angiography (CTA) during neurovascular interventions, DSA remains the gold standard worldwide.

Over the last three decades, numerous works have studied the role of DSA in both diagnosis and treatment evaluation of cardio- and cerebrovascular diseases. However, most of the existing studies were based on the visual review of image sequences by neurologists such that observations were collapsed to a simplified scale describing degree of reperfusion (Thrombolysis in Cerebral Infarction (TICI)) and recanalization (Arterial Occlusive Lesion (AOL)) after intervention. These dichotomizations are still a matter of ongoing debate [5] in the stroke community as their correlation with general outcome is limited and may also present interreader variability. There is a clear need to go beyond these manual scoring systems to obtain better evaluations for future clinical trials and endovascular devices. Although automatic TICI and AOL scores are still beyond the capabilities of current methods, automated algorithms for quantitative blood flow estimation have been developed over the last 20 years (as reviewed in [6]). They have failed so far to be translated into meaningful tools that could improve clinical practice and treatment evaluation.

In addition to its high spatial resolution, DSA holds temporal information that can be used to track the contrast agent and compute parametric perfusion parameters, such as cerebral blood volume (CBV), cerebral blood flow (CBF), mean transit time (MTT), time-to-peak (TTP), and *T*_max_, thus providing a quantitative assessment of cerebral hemodynamics. Those parameters are very useful for assessment of cerebrovascular diseases as they can render the underlying functional information more easily recognizable. In contrast with MR and CT perfusion imaging that have been studied through clinical trials (e.g., DEFUSE [7]), the value of parametric imaging from DSA has been largely underappreciated. While the idea of extracting perfusion parameters from DSA has been introduced since the 1980s in sporadic studies for CBF [8], MTT [9], TTP [10], and *T*_max_ [10, 11], it has not yet received the attention deserved by the acute stroke community. This overall lack in interest may be caused by the difficulty of real-time implementation of those algorithms on angiographic units, challenges due to the nature of the images (artifacts, vessel overlap), or perhaps failure to realize its full potential for quantitative decision support. Drawing from these observations, the overall goal of this paper is to describe how these important perfusion parameters can be extracted from the time-density curve and displayed in color-coded images that can be readily interpreted by neurologists and neurointerventionalists. After a brief historical review in Section 2.1 and a description of the dataset in Section 2.2, the paper summarizes the theory of densitometry in Section 2.3 and introduces the proposed framework in Section 2.4. The results of the experiments are presented in Section 3 and discussed in Section 4.

## 2. Methods

### 2.1. Historical and Technical Overview

Since its discovery [12], the application of X-ray for imaging purposes has greatly influenced medical diagnosis and interventions as it allows visualizing moving anatomical structures and endovascular devices. X-rays are produced by accelerating electrons emitted from a cathode towards a metal target anode using high voltage (50 kV). When directed towards the body and by passing through it, X-rays are partially absorbed and deflected, which causes attenuation of the incident beam. Various anatomical structures can be differentiated thanks to their specific level of absorption.

One of the decisive milestones of X-ray imaging was the introduction of angiography [13] which has made the visualization of blood flow within vessels possible. The acquisition of an angiogram relies on X-ray imaging of iodinated radioopaque contrast agent previously injected into the blood stream. The blood flow is observed thanks to the high level of absorption of the contrast agent.

The introduction of the image intensifier television (II-TV) that converts the incident X-rays into a visible image was an ingredient to the success of angiography. With the modernization of computers in the 1980s, it became possible to record images digitally. This led to digital radiography [2] which allows for more flexible visualization of digitally enhanced images. Digital subtraction angiography (DSA) extends digital radiography [4, 14–17] by subtracting a background image (obtained before injection of the contrast agent) from subsequent images. The purpose is to eliminate the bone and soft tissue images that would otherwise be superposed on the vessels.

Despite excellent resolution characteristics, DSA has several inherent shortcomings. First, DSA images are subjected to two major types of noise: the quantum noise due to the random nature of X-ray distribution and the noise resulting from the electronic components. In addition, the image subtraction operation amplifies the noise already present in images. To overcome this problem, noise reduction techniques can be applied. This leads to a second weakness of DSA: noise reduction algorithms are generally coded in the hardware and algorithms cannot easily be accessed or customized. Another limitation is the possible motion of the patient during the image acquisition that creates spatial blur and artifacts as the background image is not aligned to subsequent frames. Finally, visualization of the temporal information from DSA sequences is challenged by the fact that images are typically displayed in a video mode with raw grayscale frames. Only recently have commercial systems started to introduce colormaps to better visualize the temporal information held in DSA.

In summary, DSA is technological evolution of digital radiography to remove unwanted bone and soft tissue from a set of successive images. Besides the technical limitations due to the nature of X-ray imaging, DSA is associated with a computational layer that may also introduce significant inaccuracies in the presence of even minor patient motion. Because internal parameters and source images used by DSA are generally not made available by scanner manufacturers, further postprocessing is particularly challenging. Despite these limiting factors, DSA remains the gold standard used during endovascular interventions. In this study, we proposed to extend DSA by introducing a computational framework for the computation of perfusion parameters.

### 2.2. Patients Demographics and Data Acquisition

The imaging dataset used in this study to evaluate our framework was collected from patients evaluated at a single, academic comprehensive stroke center and identified with symptoms of acute ischemic stroke. The use of this dataset was approved by the local Institutional Review Board (IRB). Inclusion criteria for this study included (1) presenting symptoms suggestive of acute stroke, (2) last known well time within six hours at admission, (3) digital subtraction angiography (DSA) of the brain performed at the end of a thrombectomy procedure, and (4) final diagnosis of ischemic stroke. A total of 66 patients (median age: 68 years (IQR 53, 79)), including 35 women, satisfied the above criteria. All patients underwent thrombectomy with various success in revascularization which was determined using the Thrombolysis in Cerebral Infarction (TICI) score. The distribution of TICI scores is as follows: TICI 0 (4 patients), TICI 1 (1 patient), TICI 2a (17 patients), TICI 2b (35 patients), and TICI 3 (9 patients). Mechanical clot-retrieval devices include Trevo® (7 patients), MERCI® (17 patients), and Solitaire® (32 patients). The median NIH stroke scale (NIHSS) is 18, IQR 13, 21. The DSA scanning was performed on a Philips Allura Xper FD20® biplane using a routine timed contrast-bolus passage technique. Manual injection of Omnipaque 300 was performed at dilution of 70% (30% saline) such that 10 cc of contrast was administered intravenously at an approximate rate of 5 cm^3^/s. Image acquisition parameters vary across subjects. In the biplane acquisition setting, frames are acquired in an interleaved fashion at two standard viewpoints: anterior-posterior (AP) and lateral. The median number of frames acquired is 20 frames, IQR 17, 22, and the median peak voltage output is 95 kV, IQR 86, 104. Images sizes were all 1024 × 1024 but were acquired with different field of view.

### 2.3. Video Densitometric Theory

To derive perfusion parameters from DSA sequences by bolus tracking analysis, the concentration *C* of the contrast agent at any location must be known. It can be estimated through DSA as the intensity observed in the image is proportional to the contrast concentration [8, 18]:

(1) $$\begin{array}{c} I{\left(\;t\;\right)}_{\left(x,y\right)}=k\mu C{\left(\;t\;\right)}_{\left(x,y\right)}\rho_{\left(x,y\right)}, \end{array}$$

where *I*(*t*)_(*x*,*y*)_ is the DSA image intensity value for a given pixel (*x*, *y*) at time *t*, *μ* is the mass attenuation coefficient of the contrast agent which is proportional to the X-ray energy, *ρ*_(*x*,*y*)_ is the thickness of the vessel, *C*_(*x*,*y*)_ is the contrast concentration, and *k* is a constant that accounts for the X-ray imaging system acquisition and amplification [19].

The vessel thickness *ρ*_(*x*,*y*)_ can be computed using one of the previously described frameworks (e.g., [20]) that first applies a vessel detector based on vesselness filtering and thresholding. Centerlines are then obtained via skeletization. Finally, a perpendicular segment (computed along each point of the centerline) is used to measure the distance to the edges of the vessel and derive the thickness assuming cylindrical volume. The thickness is then applied on a cross-sectional basis to every point within the vessel using bicubic interpolation.

Animal studies [21, 22] of coronary circulation from DSA have demonstrated accurate estimation of the flow within blood vessels. Other studies [23] estimated flow related parameters from contrast time curves within the pulmonary parenchyma. In those cases, the vessel diameter within the parenchyma was too small to be measured on the image and had to be set to a constant value *ρ*_(*x*,*y*)_ = *k*_*ρ*_.

We transpose these estimation methods of the concentration-time curve within blood vessels and the brain parenchyma to DSA imaging routinely acquired during endovascular treatment of acute ischemic strokes. The goal is to extract cerebral hemodynamic parameters to quantify degree of perfusion and delay, as described in the next section.

### 2.4. Perfusion Parameters from DSA Using Bolus Tracking

Bolus tracking algorithms [24–27] are well established methods to determine flow and timing parameters of a bolus travelling from a source to a target location. This section describes the extraction process of hemodynamic indices that will provide a quantitative description of the tissue status from DSA.

From the contrast concentrations (see (1)), it is possible to estimate the CBV at any location *u* in the image by calculating the amount of contrast agent *C*_*u*_ that has passed through it with respect to the total amount of contrast measured at the feeding arterial vessel *C*_*a*_ (i.e., arterial input function (AIF)):

(2) $$\begin{array}{c} CBV=\frac{\int_{t=0}^{\infty }C_{u}\left(t\right)dt}{\int_{t=0}^{\infty }C_{a}\left(t\right)dt}. \end{array}$$

Assuming no recirculation and therefore unimodality of the contrast curves, it is common to use the peak of the contrast curve as a temporal landmark. The time taken to reach that maximum is called time-to-peak (TTP).

It can be shown that the temporal relationship between the concentration at the feeding artery *C*_*a*_ and the target tissue *C*_*u*_ can be written as

(3) $$\begin{array}{c} C_{u}\left(\;t\;\right)=C_{a}\left(\;t\;\right)⊗h\left(\;t\;\right), \end{array}$$

where ⊗ is the symbol for the convolution and *h* is the distribution of the transit times, as the contrast agent follows different paths through the vasculature. The transit times are related to the fraction of injected contrast agent still present in the vasculature at any given time *t*. This measure is described by the residue function *R*(*t*):

(4) $$\begin{array}{c} R\left(\;t\;\right)=1-\overset{t}{\underset{\tau =0}{\int }}h\left(\;\tau \;\right)d\tau . \end{array}$$

From *R*, we can establish the relation between the concentrations *C*_*u*_ and *C*_*a*_:

(5) $$\begin{array}{c} C_{u}\left(\;t\;\right)=CBF\left(\;C_{a}⊗R\;\right)\left(\;t\;\right) \end{array}$$

which indicates that the contrast concentration *C*_*u*_(*t*) in the target tissue at a given time *t* is proportional to the amount of blood passing through per unit time (i.e., CBF).

While the concentrations *C*_*a*_ and *C*_*u*_ can be estimated from DSA (see (1)), the residue function *R* and CBF require more complex computations. In practice, the concentration curves *C*_*a*_ and *C*_*u*_ are sampled at discrete time points, *t*_*j*_ ∈ [0, *N* − 1]:

(6) $$\begin{array}{c} C_{u}\left(\;t_{j}\;\right)=\Delta tCBF\overset{N-1}{\underset{i=0}{\sum }}C_{a}\left(\;t_{i}\;\right)R\left(\;t_{j}-t_{i}\;\right) \end{array}$$

which can be rewritten as a matrix-vector notation:

(7) $$\begin{array}{c} C_{u}=\Delta tCBFC_{A}R, \end{array}$$

where *C*_*u*_, *R* ∈ *ℛ* ^*N*^ and *C*_*A*_ is expanded to a Toeplitz matrix:

(8) $$\begin{array}{c} C_{A}=\left(\begin{array}{cccc} C_{a}\left(t_{0}\right) & 0 & \cdots & 0\\ C_{a}\left(t_{1}\right) & C_{a}\left(t_{0}\right) & \cdots & 0\\ \vdots & \vdots & \ddots & \vdots \\ C_{a}\left(t_{n-1}\right) & C_{a}\left(t_{n-2}\right) & \cdots & C_{a}\left(t_{0}\right) \end{array}\right). \end{array}$$

One way to recover *R* is to use singular value decomposition (SVD) of *C*_*A*_ into two orthogonal matrices, *U* and *V* ^*T*^, and a diagonal matrix, *W*, with singular values ordered descendingly in the diagonal, *C*_*A*_ = *UWV* ^*T*^. The solution is then given by

(9) $$\begin{array}{c} R=V{\hat{W}}^{-1}U^{T}C_{u}, \end{array}$$

where elements of $\hat{W}$ that are below a threshold are set to zero.

**Figure 1:**
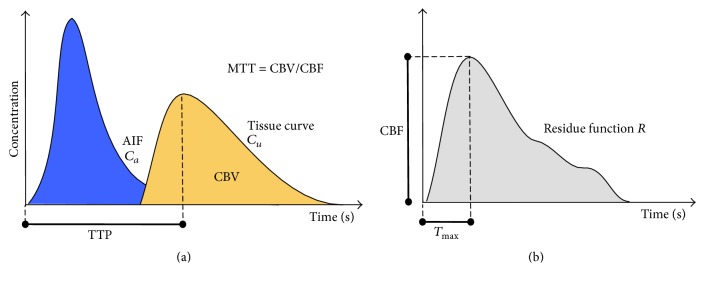
Illustration of a tissue concentration-time curve *C*_*u*_ (yellow) with respect to an arterial input function (AIF) *C*_*a*_ (blue). The deconvolution of the tissue curve *C*_*u*_ with *C*_*a*_ removes the dependence on the AIF and produces the residue function *R* (b). CBF is extracted at the maximum value reached at *T*_max_, while MTT is calculated as CBV/CBF, where CBV is determined as the area under the tissue curve (yellow). Because of the presence of arterial delays in stroke patients, the residue function is not always maximal at *t* = 0 but might be maximal after a delay (*T*_max_).

Given that max⁡(*R*) = 1, CBF is derived as the maximum of the estimated *R*, and *T*_max_ is the time to reach this maximum. Once CBF has been estimated, MTT can be derived from the central volume theorem [28], MTT = CBV/CBF. The list of parameters extracted (CBF, CBV, MTT, TTP, and *T*_max_) is illustrated in Figure 1.

### 2.5. Solving Vessel Overlap with Gamma Mixture

**Figure 2:**
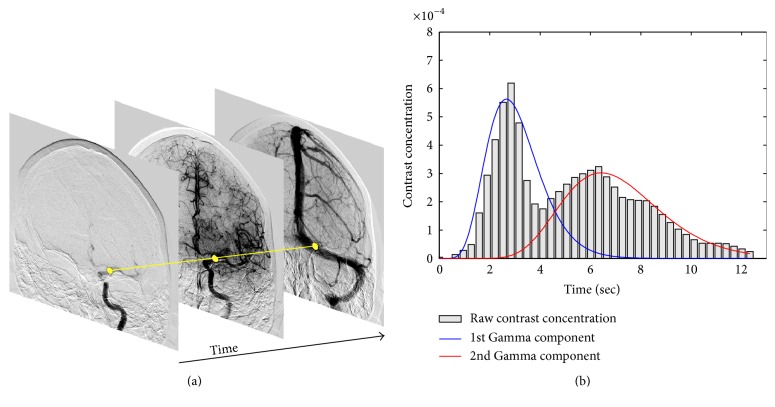
The bar graph of the contrast concentration-time curve (b) is shown for a specific location in a DSA sequence (shown in yellow on (a)). Two contrast passages can be observed in the concentration-time curve due to the overlap of the vessels. By applying the proposed method based on the EM algorithm, we are able to retrieve the individual components (represented by blue and red curves) using a Gamma mixture representation.

Overlap of the vessels may occur in biplane DSA and is one of the most challenging aspects of the estimation of perfusion parameters. This issue is illustrated in Figure 2 where a selected image location, shown as a yellow region, presents two contrast passages that lead to two peaks in the concentration-time curve. These two distributions correspond to the arterial and venous phase, respectively. The deconvolution method presented in Section 2.4 assumes unimodality of the concentration-time curve. Although it might be possible to use a previously acquired 3D model of the cerebrovasculature to delineate the vessels from the 2D projection, the direct processing of biplane DSA without any prior imaging is of great interest as other imaging modalities are not always available. To solve this problem, we suggest representing the concentration over time by a mixture of Gamma distributions that is automatically recovered at each point of the image using an expectation-maximization (EM) algorithm.

#### 2.5.1. Gamma-Variate Fitting

The Gamma-variate function is the most commonly used prior distribution to represent concentration-time curves as it has been shown to closely approximate the true contrast concentration. Drawing from the formulations present in the literature [29–31], we constrain the estimation of the concentration-time curves by assuming a minimum transit time Δ_min_ between the injection site and the brain which ensures that the maximum of the fitted distribution (which is also its inflection point) lies within the restricted domain. The density function *γ*_*α*,*β*_ is written as

(10) γα,βx=βαΓαexp−x−μβx−μα−1if x−μ≥Δmin0otherwise,

where *α*,  *β*, and *μ* are the shape, scale, and location parameters, respectively. The Gamma function Γ(*α*) is written as

(11) $$\begin{array}{c} \Gamma \left(\;\alpha \;\right)=\overset{\infty }{\underset{0}{\int }}t^{\alpha -1}{exp}^{-t}dt. \end{array}$$

The mean of the Gamma distribution is *α*/*β*. The shape of the Gamma distribution is determined by the *α* parameter, which intuitively relates to the contrast concentration variation. When *α* > 1, the distribution is bell-shaped, suggesting low heterogeneity. In the case of *α* < 1, the distribution is highly skewed which indicates high variation. This flexibility makes the distribution suitable for accommodating with different concentration-time curves as observed at different locations in the image.

#### 2.5.2. Mixture of Gamma-Variate Distributions

To capture multiple contrast passages at a given image location, we propose to represent the concentration curve over time as a mixture of Gamma-variate distributions. This assumes that the overall distribution is generated from a few Gamma components, each with its own *α* and *β* parameters. In our case, each component can be thought of as one contrast passage through one of the overlapped vessels at the current image location. Let *K* be the number of Gamma components in the mixture; the parameters of the *j*th component are denoted by *α*_*j*_ and  *β*_*j*_ and associated with the prior probability *τ*_*j*_ that a measured concentration was drawn from the current component. Parameters of the overall distribution are summarized as Θ = {*α*_*j*_, *β*_*j*_, *τ*_*j*_}, *j* = 1,…, *K*, with ∑_*j*=1_^*K*^ *τ*_*j*_ = 1, and the mixture is written as

(12) $$\begin{array}{c} M\left(\;x,\Theta \;\right)=\overset{K}{\underset{j=1}{\sum }}\tau_{j}\gamma_{\alpha_{j},\beta_{j}}\left(\;x\;\right), \end{array}$$

where *γ*_*α*_*j*_,*β*_*j*__(*x*) is the Gamma-variate distribution of the *j*th component (see (10)).

#### 2.5.3. Parameter Estimation

The optimization of the parameters Θ of the mixture is posed as a maximum likelihood estimation (MLE). The log-likelihood of parameter set Θ is obtained by approximation using a weighted sum over discrete time:

(13) $$\begin{array}{c} L\left(\;\Theta \;\right)=\overset{N}{\underset{i=1}{\sum }}\log M\left(\;x_{i},\Theta \;\right), \end{array}$$

where *i* represents a discrete time point. The parameters Θ of the model are unknown and are estimated using the expectation-maximization (EM) algorithm [32] which provides a convenient approximation in terms of an iterative maximization problem.

To be able to estimate the parameter set Θ that maximizes *ℒ*, the EM algorithm introduces an unobservable matrix *z* ∈ {0,1}^*N*×*K*^ to specify which Gamma component the *i*th observation *x*_*i*_ comes from. In the original EM algorithm, *z* is defined as a binary variable that contains 1 for the component it comes from and 0 for all the others. Here, we use the soft EM definition where *z* is continuous and can take any value between 0 and 1, such that *z* ∈ [0,1]^*N*×*K*^, and where the sum of the weights of each observed data point *i* is equal to 1, ∑_*j*=1_^*K*^ *z*_*ij*_ = 1.

The complete discrete log-likelihood becomes

(14) LΘ=∑i=1N ∑j=1Kzijlog⁡τj+C,C=∑i=1N ∑j=1Kzijlog⁡γαj,βjxi.

EM uses the log-likelihood and iterates between the two following steps.

*E-Step*. Calculate the expected value *Q*(Θ, Θ^*m*^) of the log-likelihood given current parameters Θ^*m*^, and

(15) $$\begin{array}{c} Q\left(\;\Theta ,\Theta^{m}\;\right)=\overset{N}{\underset{i=1}{\sum }}\;\overset{K}{\underset{j=1}{\sum }}z_{ij}^{m}\log\tau_{j}+C, \end{array}$$

where

(16) $$\begin{array}{c} z_{ij}^{m}=\frac{\tau_{j}^{m}\gamma_{j}\left(x_{i};\alpha_{j}^{m},\beta_{j}^{m}\right)}{M\left(x_{i},\Theta^{m}\right)}. \end{array}$$

*M-Step*. *Q*(Θ, Θ^*m*^) is maximized with respect to Θ using numerical optimization

(17) $$\begin{array}{c} \Theta^{m+1}=\underset{\Theta }{\mathrm{argmax}}\;Q\left(\;\Theta ,\Theta^{m}\;\right). \end{array}$$

The iterative procedure is executed until the convergence criterion |Θ^*m*+1^ − Θ^*m*^| < *t*_EM_ is satisfied or the maximum number of iterations reached (100). To avoid local maxima, it is repeated 5 times. The EM procedure can be performed for a different number of components *K* ∈ [1,4], for instance. The number *K* can be selected so that it minimizes the Bayesian Information Criterion (BIC) [33]. To allow for faster convergence and reduce the risk of falling into local maxima, the procedure is initialized with *k*-means algorithm.

### 2.6. Experimental Setup

This section describes the experimental protocol used in our study to evaluate the perfusion angiography framework. The proposed experiments will provide valuable insights about the following questions: Can the multivariate Gamma fitting method delineate individual contrast concentration curves in the presence of overlap and noise? How does it compare to a state-of-the-art fitting algorithm (RANSAC)? Is the computation of perfusion parameters from routinely acquired DSA feasible for assessment during endovascular interventions?

These questions are addressed by evaluating the perfusion angiography framework on two different experiments. The first experiment focuses on the estimation of the overlapped contrast concentration curves and identification of the individual components using the multivariate Gamma fitting technique (Section 2.5). To do so, we computed the average AIF concentration curves *C*_*a*1_ from 5 randomly selected patients from our dataset on which we selected a region of interest at a similar location on the intracerebral artery (ICA). The average concentration curve *C*_*a*1_ was smoothed using a Gaussian filter and interpolated to produce a set of *N* = 100 values using bicubic interpolation. The overlap was simulated by duplicating the contrast curve *C*_*a*1_ to create a vector *C*_*a*2_, shifting the duplicated vector *C*_*a*2_, and merging them into a single vector *C*_gt_, thus creating a simulated overlap between two similar contrast curves, as written as follows:

(18) Cgti=Ca1iif i−shift≤0,  ∀i∈1,NmaxCa1i,Ca2i−shiftotherwise,

where the ground truth *C*_gt_ corresponds to a multimodal contrast curve obtained from two contrast curves *C*_*a*1_ and *C*_*a*2_, such that the latter is temporally shifted. In our experiments, we produced a set of merged concentration curves by varying the shifting amount from 5 to 100, ranging from almost full to no overlap. The objective of the experiment is then to measure how accurately it can fit and retrieve the two original contrast curves *C*_*a*1_ and *C*_*a*2_ using a Gamma-variate mixture *γ*_1_,  *γ*_2_ from the merged contrast curve *C*_gt_. In addition to the evaluation of the robustness to the amount of overlap, various levels of white Gaussian noise are added to the signal, ranging from a SNR of 500 to 5.

Alternative methods to fitting Gamma distributions exist in the literature. Among them, the least squares fitting based on a discrete formulation would be possible but computationally costly. A more efficient technique is the random sample consensus (RANSAC) method [34] that has emerged as a versatile tool for robust parameter estimation in pattern recognition. It is typically used in computer vision to retrieve correspondence between images and estimate the geometric transformation matrix that relates them. The idea behind RANSAC is to estimate a large number of minimal-set fitting hypotheses. For each hypothesis, a robust score is calculated; this score is based on the alignment of the hypothesis with all points in the set. The best scoring minimal-set hypothesis is taken as the final estimate. In our experiments, a total of 300 fitting hypotheses were used and each hypothesis was made of 15 points. The accuracy of both the Gamma-variate and the RANSAC models is measured as the coefficient of determination or *R*-squared. For better estimation of the error, the process is repeated 10 times for each combination of error and overlap, and the average *R*-squared is reported.

For the second experiment, we ran the perfusion angiography on our dataset (Section 2.2) composed of DSA sequences following endovascular thrombectomy recorded on 66 acute ischemic stroke patients with MCA occlusion. The experiments are formulated such that the distribution of a given perfusion parameter across the MCA territory is averaged and studied with respect to the TICI score. Statistical measures of correlation and dispersion are extracted.

During our experiments, source DSA images of each patient are processed with perfusion angiography. The concentration-time curve of the arterial input function (AIF) *C*_*a*_ (see (2)) required for the computation of perfusion maps is obtained by extracting the average of the DSA values comprised within a region of interest (ROI) at each time point. This ROI was manually selected by a UCLA neurologist on the source DSA of each patient prior to the processing. In this study, it was set on the intracerebral artery (ICA) as an elliptical region fully included in the vessel. Note that, similar to perfusion MRI, it should be possible to detect or estimate the AIF automatically using constraints on early arrival time and maximum contrast values. However, to minimize possible source of error for the computation of perfusion parameters in this study, we chose to delineate the AIF manually. The perfusion angiography was ran using the BIC criterion to select among a maximum of two Gamma components to differentiate between the arterial and the venous phase. After processing, the following parameter maps are available; CBF, CBV_full_, CBV, MTT, TTP, and *T*_max_, where CBV_full_ is the cerebral blood flow computed over the entire cerebral cycle (including arterial and venous phases) and CBV is computed during the arterial phase only.

In order to evaluate the five perfusion parameters presented in Section 2.4  map ∈ {CBF, CBV_full_, CBV, MTT, TTP, *T*_max_}), the parameter maps need to be transformed into quantitative values *x*_map_ that can be used as input to the statistical analysis. As a preprocessing step, a UCLA neurologist (blinded to outcome and perfusion maps) delineated the MCA territory on each DSA using a template scaled down and rotated to cover the entire territory. Each perfusion parameter is then characterized using the trimmed mean of the distribution of the values within the ROI. The trimmed mean computes the average of the values comprised between the 5th and 95th percentiles:

(19) $$\begin{array}{c} x_{map}=\frac{\sum_{i}^{}\sum_{j}^{}v\left(i,j\right)}{N_{v}}, \end{array}$$

where *N*_*v*_ is the number of points included in the ROI and comprised between the 5th and 95th percentiles and *v*(*i*, *j*) is the value of the perfusion map at point [*i*, *j*]:

(20) vi,j=mapi,j;if t5<mapi,j<t95  &&  ROIi,j==10;otherwise.

We evaluate the Pearson correlation between the following pairs of variables: (CBF, TICI), (CBV, TICI), (TTP, TICI), (MTT, TICI), and (*T*_max_, TICI). To facilitate the statistical analysis, qualitative TICI scores (“0,” “1,” “2a,” “2b,” and “3”) are mapped to a continuous space, as follows: (“0,” 0); (“1,” 0.25); (“2a,” 0.5); (“2b,” 0.75); (“3,” 1).

## 3. Results

**Figure 3:**
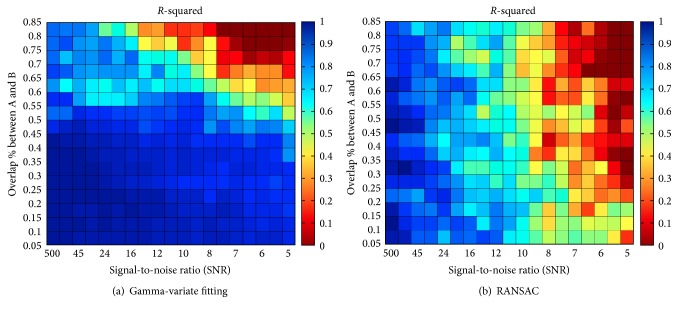
Illustration of the *R*-squared correlation coefficient between the estimated Gamma components and the ground truth for various levels of Gaussian white noise in terms of signal-to-noise ratio (SNR) and percentage of overlap between the two original components. The results are reported for the Gamma-variate method (a) and the RANSAC algorithm (b).

**Figure 4:**
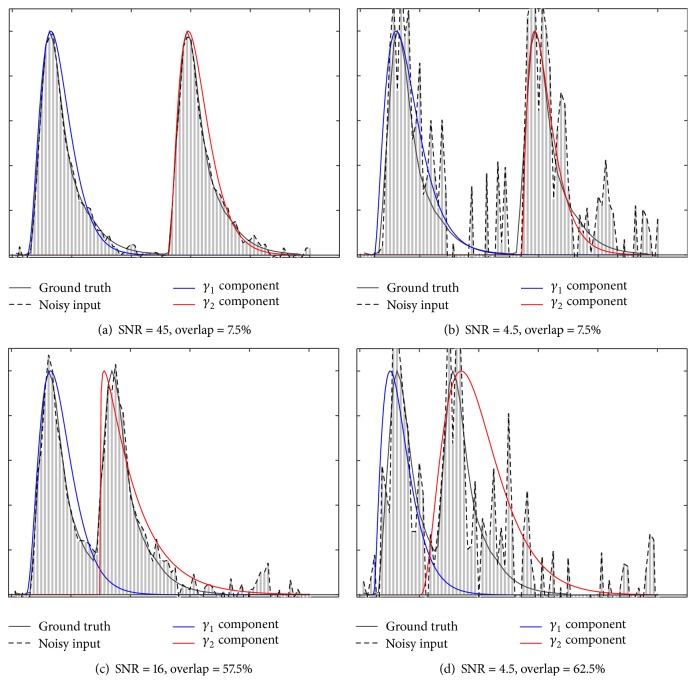
Illustration of the Gamma fitting process to recover two components for 4 different combinations of noise and overlap. Components *γ*_1_ and  *γ*_2_ are shown in blue and red and were estimated using the EM-based Gamma-variate fitting (Section 2.5) based on the noisy input depicted by the dashed line.

The results of the first experiment are reported in Figure 3 with the *R*-squared coefficient between the ground truth and the recovered mixture. It was computed for various levels of noise and degrees of overlap between two simulated contrast concentration curves within the ground truth. It can be observed that the Gamma-variate fitting framework is able to accurately retrieve the two components of the mixture in the presence of noise when the overlap is below 55%. When the overlap is greater than 55%, the accuracy decreases significantly as the noise increases. As expected, the model fails to accurately recover the two components in the presence of very high levels of noise (SNR < 8) and high percentage of overlap (>70%). Fitting results are illustrated in Figure 4 for four different combinations of overlap amount and noise levels. RANSAC recovers the components with a decent accuracy regardless of overlap until a SNR of about 10, and then the error drastically increases in the presence of higher levels of noise. In comparison, the standard estimation of TTP (without multimodal fitting) taken at the maximum of the concentration-time curve would be misplaced in half of the cases depending on which component is the highest.

In the second experiment, the perfusion angiography framework processed successfully 89% (59 out of 66) of the DSA images included in our dataset. Seven cases failed during processing due to either patient motion, short acquisition time (i.e., the DSA acquisition did not cover the entire injection cycle), poor image quality, or low temporal resolution (i.e., insufficient number of frames).

As a first observation, we noted that most of the patients included in our dataset (93%; 55 out of 59) had poor outcomes (mRS greater than or equal to 3). We also noticed that a TICI score of 2b leads to a slightly better mRS outcome than 2a. However, patients that reached a TICI score of 3 (i.e., complete reperfusion of the MCA territory) were not associated with a better outcome than 2b patients. The phenomenon of futile recanalization is similar to what has been reported in other studies [35]. Possible explanation may include increased risk of hemorrhagic transformation. NIHSS at admission is linearly correlated with mRS outcome (*r* = 0.304, *p* < 0.028). In addition, low NIHSSs (i.e., not severe) are associated with larger variations in terms of outcome.

Linear regression analysis between CBF and CBV values revealed an overall strong correlation (*r* = 0.736, *p* < 10^−12^). Both values are estimated with perfusion angiography and averaged over the entire MCA territory. CBV was computed during the arterial phase of the cycle. This is an expected result that has been shown in previous MR and CT studies of perfusion [36] and could in principle be used to identify infarcted areas from penumbra [37].

**Figure 5:**
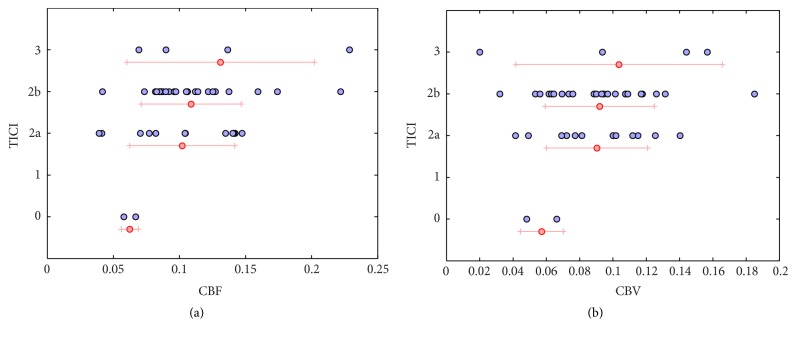
Scatter plots representing the perfusion angiography CBV and CBF versus TICI score. Average and standard deviation for specific TICI values are depicted by red lines.

Scatter plots representing the CBV and CBF perfusion angiography maps versus TICI score are illustrated in Figure 5 where each patient is depicted by a circle. The plots include CBF versus TICI (a) and CBV versus TICI (b). When plotted versus TICI, CBF shows a sign of positive correlation (*r* = 0.292, *p* < 0.064). However, low CBF is not always synonym of poor TICI score as slower flow might still lead to good revascularization and therefore a high TICI score. This may explain why larger TICI variations are observed for cases associated with low CBF. When CBV is studied with respect to TICI (Figure 5(b)), it shows weaker correlation (*r* = 0.218, *p* < 0.170). Significantly higher delays in terms of TTP were measured in the MCA territory for patients with no revascularization (TICI = 0). For other TICI grades, there was no correlation with TTP. Absence of equivalence between TICI and CBF/CBV estimated with perfusion angiography does not imply superiority of one measure to the other but rather it implies that they provide a different, perhaps complementary set of information.

**Figure 6:**
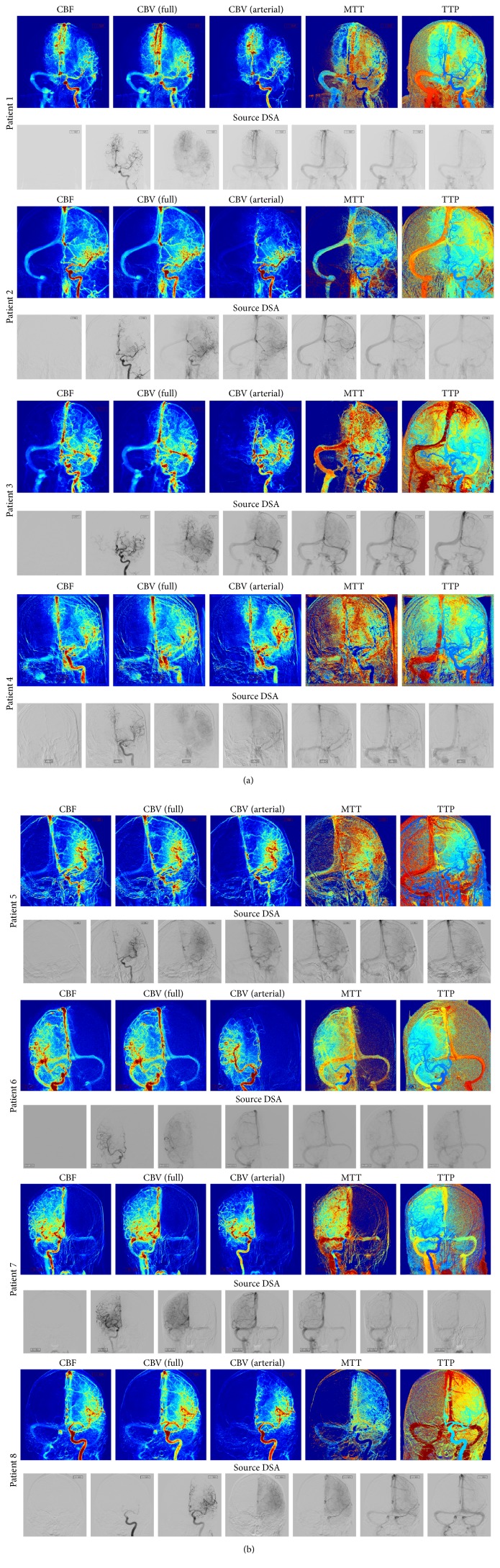
(a) Parametric maps computed for 4 patients. For each patient, the perfusion parameters are illustrated, including CBF, CBV (full) (computed over the entire arteriovenous cycle), CBV (arterial) (computed over the arterial phase), MTT, and TTP. The source DSA is shown on the bottom row of each patient. (b) Parametric maps computed for 4 patients. For each patient, the perfusion parameters are illustrated, including CBF, CBV (full) (computed over the entire arteriovenous cycle), CBV (arterial) (computed over the arterial phase), MTT, and TTP. The source DSA is shown on the bottom row of each patient.

The parametric maps computed for 8 patients are displayed in Figures 6(a) and 6(b). For each patient, the perfusion parameters are illustrated, including CBF, CBV_full_ (computed over the entire arteriovenous cycle), CBV (computed over the arterial phase), MTT, and TTP. For display purposes, each parametric map is normalized and color-coded to facilitate visualization. Red is used to show high value (↑ flow for CBF, ↑ volume for CBV, and ↑ delay for MTT and TTP), and blue is used to represent low values. In addition, the source DSA on which perfusion angiography was performed is shown on the bottom row of each case. For matter of space, a subset of seven frames were sampled and displayed for each DSA sequence.

One of the particularities of the perfusion maps is to be bidimensional; therefore, a single image region may represent different anatomical structures that overlap across that region. Despite this limitation, these maps provide fine detail as they match the original spatial resolution of the DSA (1024 × 1024 in our dataset). When reviewed side by side, CBF, CBF, and TTP maps may help the expert eye to differentiate between antegrade and collateral flow and identify risk of hemorrhage, perfusion deficit, delay, and flow stagnation. The computation of the perfusion parameters for a single patient took 21 seconds. In principle, faster execution times can be obtained as the estimation of the perfusion parameters can be parallelized.

## 4. Discussion

There is an overt need to provide imaging-based decision support to better guide and accelerate endovascular interventions in acute stroke. Among the available imaging techniques, DSA is a method of choice to visualize blood flow and guide endovascular interventions. Biplane DSA provides high-resolution spatiotemporal images that have mostly been used qualitatively through the manual review of raw grayscaled video. The interpretation of DSA images could benefit from color-coded perfusion parameters that would enable the visualization of hemodynamic features that are not directly visible on source angiograms and allow for refined decisions without any delay in care, added X-ray exposure, or higher dose of contrast agent.

We have introduced in this paper a computational framework for the extraction of quantitative perfusion parameters from routine DSA. Similar to CT/MR perfusion, our approach uses a deconvolution technique to derive CBF, CBV, MTT, TTP, and *T*_max_. A novel computational solution based on multimodal fitting was introduced to deal with overlap of the vessels. This study has demonstrated that routinely acquired DSA can be used to derive perfusion parameters that are similar in spirit to the ones obtained from CT/MR perfusion. However, the interpretation perfusion DSA is different due to the nature of the view (frontal or lateral) and the overlap of several brain structures within a given location.

Taking a step back, it is clear that neuroimaging provides neurologists and neurointerventionalists with an immense source of information for guidance in clinical decision-making. Yet, perhaps because of the abundance of information held in those images, their use remains suboptimal. At UCLA, for example, the following modalities can be acquired: magnetic resonance imaging/angiography (MRI/MRA), diffusion/perfusion-weighted MRI, computed tomography/tomographic angiography (CT/CTA), perfusion CT, and digital subtraction angiography (DSA). Broadly speaking, these images offer different insights and mirror different steps of the therapy. Neuroimaging is used before treatment to classify the stroke using lesion size, tissue at risk, and involved vascular territory. This allows identifying stroke patients who can benefit the most from a specific treatment strategy and outweigh its potential risks. DSA images are acquired during therapy for decision-making. These iterative landmarks can be used to evaluate the degree of reperfusion and recanalization by visual scoring. Beyond the acute phase, neuroimaging is helpful in evaluating recovery and guiding other management strategies such as the augmentation of cerebral perfusion and reduction of mass effects from hemorrhage. Validation of perfusion angiography for estimation of hypoperfusion volume or degree of recanalization and reperfusion during endovascular interventions would be of great interest.

The need for neuroimaging insight is triggered by the complexity of personalized treatment and variability of stroke outcomes. The patient population in acute ischemic stroke is incredibly heterogeneous; it presents a wide variety of outcomes and responses to treatment. For example, although the degree of recanalization correlates favorably with outcome, the risk of death remains stable. In addition, while the time from symptoms onset also correlates with outcome on average, it is not rare to observe that late recanalizers do better than early ones. These paradoxical observations can be linked to several factors such as blood pressure, NIH Stroke Scale (NIHSS), or age, but their individual predictive value is too weak for supporting prospective clinical decisions. The presence of collateral circulation beyond the site of occlusion may also be decisive as it could sustain tissue viability until recanalization occurs; however, its presence largely varies across patients. Therefore, careful patient selection for endovascular intervention based on collateral circulation and tissue status is key to tailor interventions and improve outcomes. Currently, collateral flow is evaluated on DSA but remains challenged by the lack of quantitative measure. Automatic evaluation of collateral flow and revascularization may be possible from perfusion angiography and should be considered for future studies.

### 4.1. Challenges and Limitations

The proposed framework holds several limitations due to the bias existing in the dataset studied, the nature of the source images, and other technical challenges related to the computation and acquisition. We discuss in this section these limitations and how they might affect the results obtained and could potentially be tackled in future studies.

A limiting factor of the study is that the dataset used in our experiments was rather small and not evenly distributed across degree of revascularization. Only five patients had poor TICI scores of 0 or 1. Conversely, mRS outcome was poor for most of the patients. Four patients fell within the range of mRS ∈[0,2]. Although the study of such a dataset can provide a proof of concept and applicability of the techniques, drawing conclusions or guidelines from the statistical analysis and the generalization power of specific perfusion parameters at the population level would require a larger, multicenter dataset. Such a study could provide more reliable estimates and possible relationship with outcome.

On the technical side, phantom calibration would in principle be required to obtain contrast curves that are generalizable across patients and hardware configuration. Because the study proposed in this paper was performed retrospectively on routinely acquired DSA, calibration values were not available. This lack of normalization has likely introduced errors in the estimated parameters. A prospective animal study with phantom calibration would be appropriate to test the accuracy of the parameter maps (especially CBF). In addition, vessel thickness was not considered during the computation of the perfusion maps. Because of this, it is likely that flow may have been incorrectly estimated in large vessel area. It should be possible to solve this problem by coupling the computation of the perfusion parameters with an automatic vessel detector that could extract the vessel diameter along the cerebrovasculature.

The time resolution of the DSA sequence (i.e., frame rate of the acquisition) has a great impact on the quality of the perfusion parameters. When the number of frames is too low, it becomes very difficult to delineate the different vessels when an overlap occurs. In addition, the estimation of CBV and TTP becomes approximative. Unlike CT and MRI where the time interval between each acquisition is kept constant, DSA sequences are acquired with a varying frame sampling rate. Therefore, the set of points of the contrast curve needs to be resampled and interpolated. In our framework, cubic spline interpolation was used and the interval was chosen as the minimum time interval observed between two successive frames in the current acquisition. Systematic tests should be performed to evaluate the sensitivity of the estimation of the perfusion parameters with respect to the frame rate.

There is a margin for improvement of the perfusion angiography framework by tackling these limitations. One of the most promising research directions would be to perform a comparative analysis to test equivalence of perfusion parameters estimated from DSA using perfusion angiography to the one obtained via MR or CT perfusion.

## 5. Conclusion

We have introduced in this paper perfusion angiography, a methodological framework for the quantitative analysis and visualization of blood flow parameters from DSA images. The parameters, including cerebral blood flow (CBF) and cerebral blood volume (CBV), mean transit time (MTT), time-to-peak (TTP), and *T*_max_, were reliably estimated using a bolus tracking method based on the deconvolution of the time-density curve on a pixel-by-pixel basis.

Although further study on a larger dataset would be necessary to establish statistical correspondence with outcome and TICI score and to provide comparative analysis with estimated parameter maps obtained with MR and CT perfusion, the proposed imaging tool provides unique insights into flow mechanisms that cannot be observed directly in DSA sequences and may be used to quantify perfusion impact of endovascular interventions.
